# The Rhomboid Protease GlpG Promotes the Persistence of Extraintestinal Pathogenic Escherichia coli within the Gut

**DOI:** 10.1128/IAI.00866-16

**Published:** 2017-05-23

**Authors:** Colin W. Russell, Amanda C. Richards, Alexander S. Chang, Matthew A. Mulvey

**Affiliations:** Division of Microbiology and Immunology, Department of Pathology, University of Utah, Salt Lake City, Utah, USA; University of Massachusetts Medical School

**Keywords:** ExPEC, UPEC, beta-oxidation, fatty acids, glycerol degradation, gut, intestinal, mucus, urinary tract infection, uropathogenic

## Abstract

Extraintestinal pathogenic Escherichia coli (ExPEC) strains are typically benign within the mammalian gut but can disperse to extraintestinal sites to cause diseases like urinary tract infections and sepsis. As occupation of the intestinal tract is often a prerequisite for ExPEC-mediated pathogenesis, we set out to understand how ExPEC colonizes this niche. A screen using transposon sequencing (Tn-seq) was performed to search for genes within ExPEC isolate F11 that are important for growth in intestinal mucus, which is thought to be a major source of nutrients for E. coli in the gut. Multiple genes that contribute to ExPEC fitness in mucus broth were identified, with genes that are directly or indirectly associated with fatty acid beta-oxidation pathways being especially important. One of the identified mucus-specific fitness genes encodes the rhomboid protease GlpG. *In vitro*, we found that the disruption of *glpG* had polar effects on the downstream gene *glpR*, which encodes a transcriptional repressor of factors that catalyze glycerol degradation. Mutation of either *glpG* or *glpR* impaired ExPEC growth in mucus and on plates containing the long-chain fatty acid oleate as the sole carbon source. In contrast, in a mouse gut colonization model in which the natural microbiota is unperturbed, the disruption of *glpG* but not *glpR* significantly reduced ExPEC survival. This work reveals a novel biological role for a rhomboid protease and highlights new avenues for defining mechanisms by which ExPEC strains colonize the mammalian gastrointestinal tract.

## INTRODUCTION

Extraintestinal pathogenic Escherichia coli (ExPEC) strains are a group of strains that can colonize the mammalian gut without causing any overt pathology. However, ExPEC strains can spread from the gut to extraintestinal niches, such as the urinary tract, the meninges, and the bloodstream, and subsequently induce disease (1, 2). Urinary tract infections (UTIs) are most often caused by ExPEC bacteria, are typically resolved with antibiotic treatment, and are usually not life-threatening, especially when infection is limited to the bladder (1). However, UTIs are quite prevalent, with half of all women requiring treatment for a UTI during their lifetime (3) and many women suffering from recurring UTIs (4). This prevalence contributes to a high economic burden, with over $2 billion being spent to treat bladder infections each year in the United States (5). In addition to the urinary tract, ExPEC bacteria can infect the bloodstream and cause severe disease, especially in patients with weakened immune responses. For example, in the context of early-onset neonatal sepsis, ExPEC is the second leading etiological agent and the primary cause of mortality (6). The concern surrounding the spectrum and prevalence of infections caused by ExPEC is compounded by the increasing prevalence of antibiotic-resistant ExPEC strains seen across the globe (7). In order to combat ExPEC more efficiently, it is critical that we understand the ExPEC life cycle in greater depth.

There are many indications that the gut may act as a reservoir from which ExPEC can disseminate and cause disease. For example, during acute UTI, the same strain can frequently be isolated from both the patient's urine and the patient's feces (8–10), and in most of these instances the uropathogen is the predominant fecal E. coli strain (8). ExPEC can also translocate directly from the gut to the bloodstream, especially in cancer patients. Indeed, ExPEC is the most common Gram-negative bacterium isolated from the blood of bacteremic cancer patients, who have reduced gut barrier functions due to immunosuppression and disruption of the intestinal mucosa (11). In addition to spreading from the gut to an extraintestinal site within a single individual, ExPEC can spread from one person to another, as in the case of neonatal sepsis, in which the newborn is often infected by an ExPEC strain originating from the mother (12). Therefore, although ExPEC is not known to cause serious disease in the gastrointestinal tract, an understanding of the gut colonization process could be helpful for developing future therapeutic options to combat extraintestinal infections.

One element that may be key to gut colonization by ExPEC is the intestinal mucus layer, which functions to separate the host epithelium from the bulk of the microbiota (13). In addition to acting as a barrier, the mucus layer can supply nutrients and points of attachment for some microbes (14). On the basis of observations made with streptomycin-treated mouse models of gut colonization, the mucus layer appears to be a major site of colonization for E. coli commensal strains (15, 16). These strains often grow *in vitro* in crude cecal mucus but not in the cecal lumenal contents, in which mucus is sparse (16, 17). There appears to be an association between the ability of a strain to grow *in vitro* in cecal mucus and the ability of that strain to colonize the mouse gut (16–18), although this is not always a perfect correlation (19).

To better understand how ExPEC colonizes the gut, we set out to document the genes that are critical for growth in mucus *in vitro* by utilizing transposon sequencing (Tn-seq), a high-throughput method for determining the contribution of each bacterial locus to fitness under a given condition (20). We found several genes that were important for ExPEC growth in mucus but not under the glucose-control condition. Many of these mucus-specific fitness genes are linked with the metabolism of fatty acids, and one gene, which encodes the rhomboid protease GlpG, was found to promote ExPEC survival within the mouse intestinal tract in the presence of the intact natural microbiota.

## RESULTS

### A Tn-seq screen for ExPEC genes that are important for growth on intestinal mucus uncovers a role for beta-oxidation.

**FIG 1:**
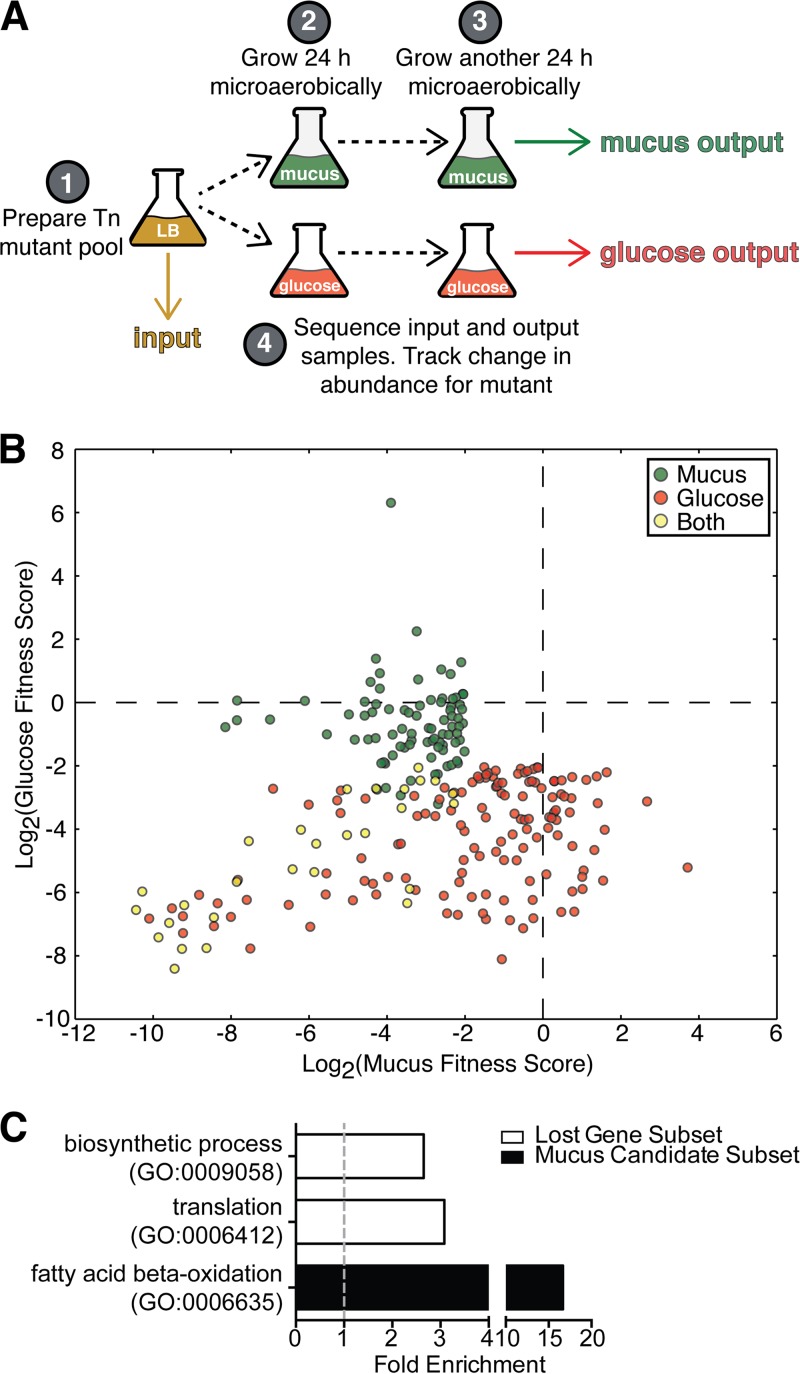
Tn-seq uncovers a role for beta-oxidation in mucus metabolism by ExPEC. (A) A schematic describing the Tn-seq experiment. An F11 transposon library was expanded aerobically in LB broth overnight and then subcultured 1:100 into a modified M9 medium supplemented with either mucus or glucose. The bacteria were then grown for 24 h microaerobically, at which point they were subcultured 1:100 into fresh medium and grown for another 24 h microaerobically. This procedure was repeated for 3 independently derived transposon mutant pools. Samples of bacterial cells were taken from the LB broth at the beginning of the experiment (input) and from the mucus and glucose media after 48 h of growth (mucus output and glucose output, respectively). These input and output samples were then prepared for sequencing to both map and quantify the transposon insertion sites. The abundance of each insertion site in the output and input samples was used to derive fitness scores. The mutant fitness scores for the three samples were pooled and used to calculate a fitness score for each gene under both the glucose and mucus conditions. (B) Candidate genes that had a fitness score of −2.0 or less (at least a 4-fold reduction in fitness) and a *P* value of ≤0.05 under mucus broth, glucose broth, or both conditions. (C) Candidate genes were analyzed via GO analysis using PANTHER. Those genes that were not detected in the input sample (Lost Gene Subset), likely due to their essential nature, were also analyzed. Those GO categories that were significantly enriched in the lost gene subset and the mucus candidate subset compared to their levels in the F11 genome are graphed.

As E. coli often associates with gastrointestinal mucus (15, 16) and since the mucus layer is known to be a source of nutrients for the microbiota (14), we reasoned that ExPEC genes required for the metabolism of intestinal mucus may also be critical for gut colonization. Therefore, a Tn-seq screen was devised in order to uncover genes important for mucus metabolism *in vitro* (Fig. 1A). Three independently derived mutant pools (21) were first expanded in Luria-Bertani (LB) broth and then subcultured into minimal medium supplemented with either glucose or porcine gastric mucus. The bacteria were then grown for 24 h microaerobically to mimic the gut environment. A second round of 24 h of growth was performed in order to increase the stringency of the screen. After a total of 48 h of microaerobic growth in minimal medium, the mutant populations were collected and the transposon insertion sites were sequenced in parallel with the input population. The abundance of individual mutants in the output and input pools were then compared, and a fitness score was derived for each mutant. After normalization and filtering (see Materials and Methods), each fitness score was calculated as log_2_(mutant abundance in output/mutant abundance in input). The median value of the various insertion site fitness scores for a given gene was defined as the fitness score for that gene.

There was a total of 24,822 transposon insertion sites for which a fitness score was determined under both the mucus and glucose conditions. On average, there was one transposon insertion detected for every 210 nucleotides. This coverage allowed a fitness score to be calculated for 3,804 genes (see Data Set S1 in the supplemental material). In order to create a list of candidate genes that contribute to fitness in either the mucus broth, the glucose broth, or both, several cutoffs were used. Only genes with a fitness score of −2.0 or lower (i.e., a minimum of a 4-fold reduction of mutant abundance) and a *P* value of less than 0.05 were included in the candidate list (Fig. 1B). These thresholds produced a list of 112 candidate mucus-specific fitness genes, 171 glucose-specific fitness genes, and 28 genes that appeared to be important for ExPEC growth in both types of media (Data Set S1).

Several genes of note were found to be fitness candidates in both the mucus and glucose broth, likely due to their essentiality for microaerobiosis, a feature that both growth conditions had in common. For example, *rqlI* was found to be a candidate gene in both media, with a fitness score in mucus of −5.8 and a score of −4.5 in glucose. This is unsurprising, as the Δ*rqlI* mutant exhibits a growth defect under low-oxygen conditions due to the inherent toxicity of the helicase RqlH in the absence of its binding partner, RqlI (22). Another candidate gene that is known to have critical functions under low-oxygen conditions was *arcB*, which had a fitness score of −3.5 in mucus and a score of −6.3 in glucose. ArcB is a sensor histidine kinase that forms a two-component system with ArcA (23), a transcriptional regulator that is important for proper microaerobic metabolism (24). Other candidate fitness genes that were identified under both growth conditions included *nuoF*, *nuoG*, and *nuoL*, which encode proteins that are a part of NADH dehydrogenase 1 (NDH-1). Five other genes encoding NDH-1 components (*nuoC*, *nuoI*, *nuoJ*, *nuoM*, and *nuoN*) were candidate fitness genes in glucose broth, but few or no transposon insertions within these genes were detected following growth in the mucus broth, making it difficult to assess their importance in that medium. NDH-1 functions as part of the electron transport chain in the transfer of electrons from NADH to the quinone pool and is especially efficient when fumarate or dimethyl sulfoxide instead of oxygen is used as the terminal electron acceptor (25). The prevalence of NDH-1 components in the list of candidate genes, along with the presence of *rqlI* and *arcB*, highlights the prominence of microaerobic metabolism in this Tn-seq experiment.

To determine in an unbiased fashion if common pathways or functions were enriched in any of these candidate gene groups, gene ontology (GO) analysis was performed using protein analysis through evolutionary relationships (PANTHER) (26). As a control for the GO analysis, a gene subset which included only genes that were not detected in the input sample, likely due to their essential nature, was also analyzed. This essential gene subset was found to be enriched for the translation and biosynthetic process GO categories, both of which are made up of genes that are critical for basic functions, such as translation, transcription, and replication (Fig. 1C). No GO enrichments were observed in the glucose candidate gene subset. However, the fatty acid beta-oxidation GO category was enriched in the mucus candidate gene subset (Fig. 1C), indicating that genes related to beta-oxidation are found under the mucus condition more often than would be expected by chance. These genes included *fadL*, *fadE*, *fadB*, and *fadJ*, all of which have been shown to be important for the uptake and metabolism of long-chain fatty acids (LCFAs) via beta-oxidation (27, 28). Beta-oxidation results in the production of several acetyl coenzyme A (acetyl-CoA) molecules which can then enter into the citric acid cycle. These data indicate that the bacteria metabolized long-chain fatty acids during the Tn-seq mucus experiment.

### Many of the candidate fitness genes are plasmid encoded.

**FIG 2:**
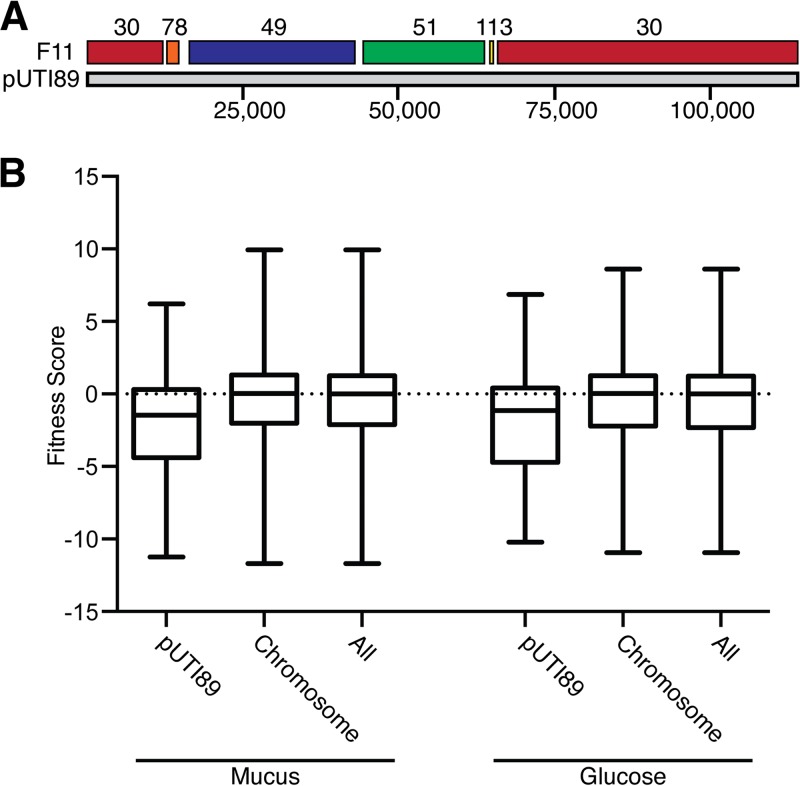
Transposon insertions in pUTI89 reduce fitness *in vitro*. (A) A schematic of the F11 contigs that align with pUTI89. The F11 contig numbers are indicated at the top, while the numbers of base pairs are noted below. (B) Range of fitness scores determined following growth in mucus or glucose broth for plasmid (pUTI89)-associated, chromosomal, and total transposon insertion sites. Bars indicate median values, with whiskers extending to minimum and maximum values.

In both mucus and glucose broth, there was a striking predominance of F11 candidate fitness genes that are likely located on a large plasmid that is very similar to the pUTI89 plasmid carried by the reference cystitis isolate UTI89 (29). There were five F11 contigs that closely aligned to the pUTI89 plasmid at the nucleotide level, with relatively small gaps existing between contigs (Fig. 2A). This plasmid is 114 kb in size and expresses genes for conjugation machinery, some genes that are associated with pathogens such as enteroinvasive E. coli (EIEC), and various other predicted open reading frames (29). Several genes located within these 5 plasmid-associated contigs met the requirements of candidate fitness genes in both mucus and glucose broth. Expanding the analysis to all insertion sites found on pUTI89 indicated that transposon insertions in the plasmid led to reduced bacterial fitness in general (Fig. 2B). The median fitness score of the plasmid-localized insertion sites was −1.46 in the mucus broth, in comparison with 0.04 for chromosomal insertion sites. Similar results were observed in glucose broth, where the median fitness scores were −1.14 for the plasmid-associated insertion sites and 0.05 for the chromosomal insertion sites. These data suggest that carriage of a fully functional pUTI89 plasmid had a notable impact on the fitness of F11 in the Tn-seq experiment.

### The *fadL*, *fbp*, and *glpG* genes are important for growth in mucus.

**FIG 3:**
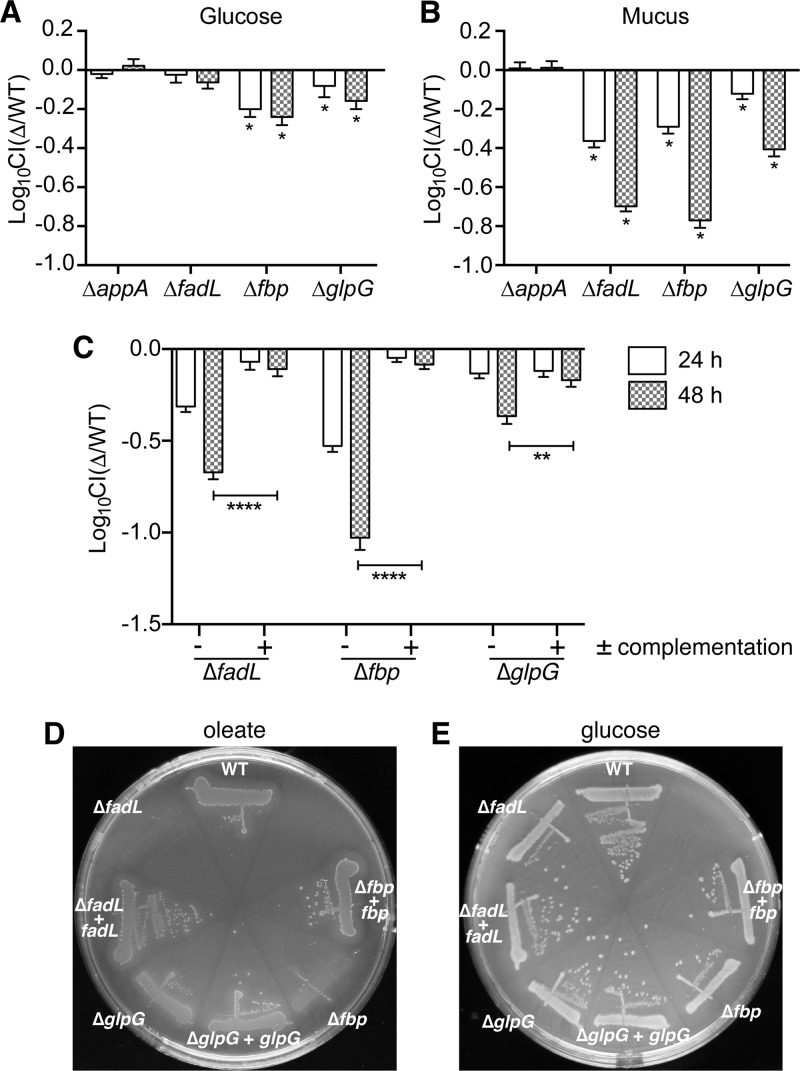
Mucus-specific candidate fitness genes are important for growth on the LCFA oleate. Four genes were chosen from the Tn-seq mucus-specific candidate fitness gene list, disrupted, and competed against the WT during microaerobic growth in M9-glucose broth (A) or M9-mucus broth (B). WT strain F11 and each mutant strain were mixed 1:1 and grown microaerobically for 24 h in the indicated medium and then subcultured 1:100 and grown for another 24 h. The titers in the cultures were determined at both the 24- and 48-h time points. The log_10_ CI was calculated by dividing the mutant titers by the WT titers (Δ/WT). *, the CI was significantly different from 0 by the one-sample *t* test (*P* ≤ 0.05). (B) Bacteria were grown and titers were determined as described in the legend to panel A, except that the bacteria were grown in M9-mucus medium. *, the CI was significantly different from 0 by the one-sample *t* test (*P* ≤ 0.05). (C) Mutant strains that exhibited defects in M9-mucus broth were complemented with plasmids that expressed the specified target genes under the control of their native promoters (F11 Δ*fadL* was complemented with pCWR37, the Δ*fbp* mutant was complemented with pCWR38, and the Δ*glpG* mutant was complemented with pCWR39). The graph shows the results from competitive assays between the WT strain carrying the empty vector pCWR40 and mutant strains that were either complemented or transformed with pCWR40. *P* values were determined by an unpaired Student's *t* test. **, *P* ≤ 0.01; ****, *P* ≤ 0.0001. The bars in panels A to C indicate mean values ± SEMs from three independent experiments performed in triplicate. (D and E) The Δ*fadL*, Δ*fbp*, and Δ*glpG* mutants carrying either an empty vector control or a complementation plasmid, as described in the legend to panel C, were streaked onto agar plates containing oleate (D) or glucose (E) as the sole carbon source.

The genes *appA*, *fadL*, *fbp*, and *glpG* were chosen from the mucus-specific candidate list for further analysis. Each of these genes was knocked out and tested in competition with the wild-type (WT) strain in growth assays using mucus or glucose broth. AppA is a periplasmic phosphatase whose expression is increased under anaerobic conditions (30). The *fadL* gene encodes the outer membrane-localized transporter of LCFAs (31). Fbp mediates the rate-limiting step of gluconeogenesis, which is important for several biosynthetic pathways when glucose is absent (32). Finally, *glpG* is a part of the *glpEGR* operon and encodes a membrane-localized rhomboid protease whose natural substrate(s) is unknown (33). When each of these mutants was competed against WT strain F11 using *in vitro* growth assays, the Δ*appA* strain did not exhibit a growth defect, whereas the others did (Fig. 3A and B). The Δ*fadL* mutant demonstrated the strongest defect, having a competitive index (CI) near −0.7 (about a 5-fold decrease in fitness relative to that of the WT strain) after 48 h in mucus broth. However, in glucose broth, the Δ*fadL* mutant had a CI that did not differ significantly from 0. The Δ*fbp* and Δ*glpG* mutants grew markedly worse than the WT strain in both glucose and mucus broth, but the defects were more pronounced in mucus broth (Fig. 3A and B). The Δ*fadL*, Δ*fbp*, and Δ*glpG* mutants grew similarly to the WT strain when their respective genes or operons were restored on a plasmid (Fig. 3C), further solidifying the roles of these genes in promoting F11 growth in mucus broth.

Disruptions to the beta-oxidation pathway lead to reduced growth on minimal medium plates supplemented with the LCFA oleate (31). As expected, the Δ*fadL* mutant grew poorly on oleate but was rescued when the *fadL* gene was included on a plasmid (Fig. 3D). Interestingly, the Δ*fbp* strain followed a pattern similar to that followed by the Δ*fadL* mutant on the oleate plates, whereas the Δ*glpG* strain exhibited a subtler growth defect (Fig. 3D). Neither Fbp nor GlpG has a previously described connection to beta-oxidation. Importantly, all of the strains grew similarly on agar plates supplemented with glucose (Fig. 3E).

### Mutation of *glpG* has polar effects on the downstream gene *glpR*.

**FIG 4:**
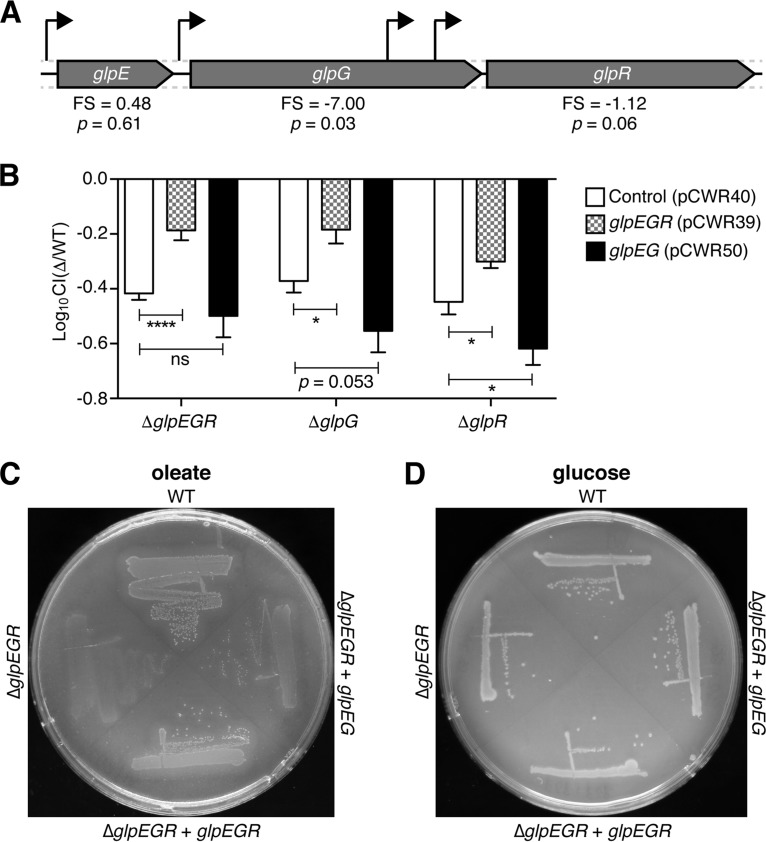
The Δ*glpG* mutant exhibits a defect in the downstream gene *glpR*. (A) A schematic of the *glpEGR* operon, with promoter locations indicated by black arrows. Below the operon are listed the fitness scores (FS; log_2_ scale) and *P* values for each gene in mucus broth determined by Tn-seq. (B) Each *glp* mutant carrying the empty vector control (pCWR40), a plasmid expressing *glpEGR* (pCWR39), or a plasmid expressing *glpEG* (pCWR50) was grown in competition with WT strain F11 carrying the control plasmid. The bacteria were grown in M9-mucus broth for 24 h microaerobically and then subcultured into fresh medium and grown for an additional 24 h. After 48 h of growth, the titer of each culture was determined to enumerate WT and mutant levels. *P* values were determined by an unpaired Student's *t* test. *, *P* ≤ 0.05; ****, *P* ≤ 0.0001; ns, not significant. Bars indicate mean values ± SEMs from three independent experiments performed in triplicate. (C and D) The WT and Δ*glpEGR* mutant strains carrying either the empty vector control or the *glpEGR* or *glpEG* expression construct, as indicated, were grown on minimal medium agar containing oleate (C) or glucose (D) as the sole source of carbon.

The *glpG* gene is the second gene of a three-gene operon that includes *glpE* and *glpR* (Fig. 4A). GlpE is a thiosulfate sulfurtransferase which is capable of transferring sulfur from thiosulfate to a recipient compound, such as thioredoxin, but its biological role is not known (34). GlpR is a transcriptional repressor of several genes that are involved with glycerol degradation (35). There are multiple promoters that drive expression of the genes in the *glpEGR* operon, including two promoters inside *glpG* that influence the transcription of *glpR* (36). It is therefore possible that transposon insertions into *glpG* or replacement of *glpG* with a resistance marker can have polar effects on *glpR*, disrupting its function. To test this possibility, Δ*glpEGR*, Δ*glpG*, and Δ*glpR* mutants were transformed with a plasmid expressing either the entire *glpEGR* operon or simply *glpEG*. Each of the mutants was then competed against the WT strain in a mucus growth assay (Fig. 4B). At 24 h, all of the mutants carrying the empty vector control exhibited a similar growth defect, with a CI of about −0.4. With the addition of the *glpEGR* expression plasmid, the fitness of each mutant notably improved. In contrast, the *glpEG* expression plasmid appeared to worsen the *glp* mutant defects. This pattern of growth was mirrored on oleate plates for the Δ*glpEGR* strain (Fig. 4C), where growth of the mutant was rescued by addition of the *glpEGR* plasmid but not by the empty vector control or *glpEG* expression plasmid. In contrast, all of the strains grew equally well on glucose medium (Fig. 4D). Altogether, these data suggest that the reduced ability of the various *glp* mutants to grow using mucus or oleate is due to disruption of *glpR* activity.

### Growth of the *glp* mutants on oleate is rescued by addition of G3P.

As GlpR is a transcriptional repressor, its absence leads to hyperactivation of the glycerol degradation pathway, which can deplete intermediates such as glycerol-3-phosphate (G3P). G3P can be further modified in this pathway to dihydroxyacetone phosphate (DHAP), which can then be utilized in glycolysis or gluconeogenesis (37). In addition, G3P is an important starting substrate for membrane biogenesis (38). Therefore, the depletion of G3P as a consequence of *glpR* disruption could lead to multiple detrimental downstream effects. Indeed, it was previously observed that a Δ*glpR* mutant defect could be rescued by the addition of exogenous G3P (39).

**FIG 5:**
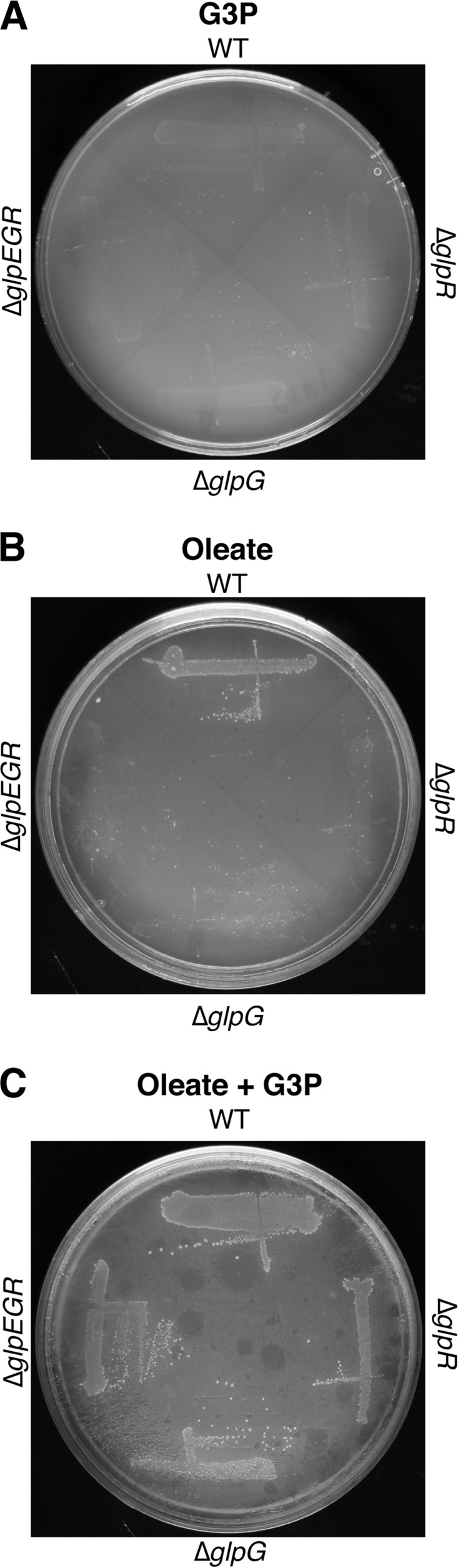
Exogenous glycerol-3-phosphate rescues growth of the Δ*glp* mutant on oleate plates. The WT, Δ*glpR*, Δ*glpG*, and Δ*glpEGR* strains were streaked onto minimal medium plates supplemented with glycerol-3-phosphate (A), oleate (B), or both glycerol-3-phosphate and oleate (C) and incubated at 37°C.

To test whether the poor growth of the F11 Δ*glpR*, Δ*glpG*, and Δ*glpEGR* strains on oleate could be rescued by the addition of G3P, the mutants were plated on minimal medium agar with or without G3P and/or oleate. None of the strains grew on control plates that were supplemented with G3P alone (Fig. 5A), and all three *glp* mutants grew markedly slower than WT strain F11 on oleate-supplemented plates (Fig. 5B). However, when G3P was added in combination with oleate, the mutant and WT strains grew similarly (Fig. 5C). These data indicate that the growth of the *glp* mutants on oleate can be rescued by the addition of exogenous G3P, suggesting that disruption of the *glp* operon causes the depletion of G3P, which in turn affects the ability of F11 to utilize LCFAs like oleate.

### GlpG is required for gut colonization, whereas FadL, Fbp, and GlpR are not.

**FIG 6:**
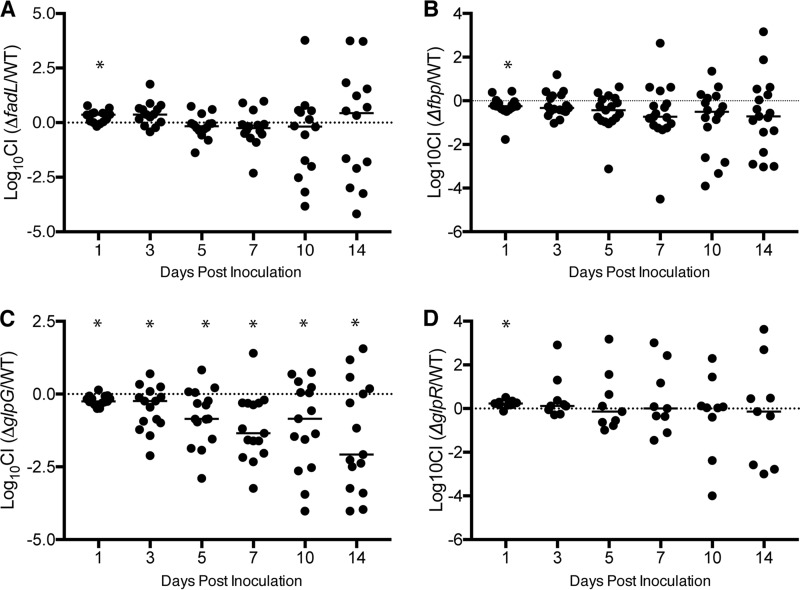
The Δ*glpG* mutant has reduced fitness during mouse gut colonization. (A to D) WT and mutant bacteria were mixed 1:1 and then delivered into mice via oral gavage at a dose of ∼10^9^ CFU. At the indicated time points postinoculation, feces were collected, homogenized, and serially diluted onto LB agar plates containing either kanamycin (to select for the WT strain) or chloramphenicol (to select for the mutant strains). Graphs show the log_10_ CI for each mouse at each time point following gavage with WT strain F11 in competition with F11 Δ*fadL* (A), F11 Δ*fbp* (B), F11 Δ*glpG* (C), or F11 Δ*glpR* (D). *, *P* ≤ 0.05 using a one-sample *t* test with the hypothetical mean set to 0, with corrections for multiple comparisons being made using the Hochberg procedure. Data are for 9 to 17 mice, and data from three independent experiments were pooled.

As the mucus layer is thought to be a source of nutrients for E. coli during gut colonization and since the Δ*fadL*, Δ*fbp*, and Δ*glpG* mutants exhibited notable growth defects in mucus broth and on oleate plates, we tested the ability of each of these mutants to colonize and persist within the mouse gut. Each mutant was competed 1:1 against WT bacteria in mouse gut colonization assays. When either the Δ*fadL* or Δ*fbp* mutant was competed against WT strain F11, the median CI was relatively close to 0 throughout the experiment (Fig. 6A and B). In contrast, when the Δ*glpG* mutant was competed against the WT strain, the median CI dropped to −1.35 by day 7 and fell further to −2.08 by day 14, representing more than a 120-fold reduction in mutant bacterial numbers relative to WT titers (Fig. 6C). These data demonstrate that disruption of *glpG* significantly reduces bacterial fitness in the gut, whereas mutation of either *fadL* or *fbp* has little effect. Of note, in these assays the total ExPEC levels (WT and mutant) remained fairly steady at ∼10^6^ CFU/g feces at each time point examined, even when the CI values were highly variable.

To assess if the defects observed with the Δ*glpG* mutant were due to polar effects on *glpR*, as is the case during growth in mucus broth and on oleate plates, we also competed F11 Δ*glpR* against the WT strain in our gut colonization assay (Fig. 6D). Interestingly, the Δ*glpR* mutant did not phenocopy the Δ*glpG* strain, exhibiting a median CI of 0.23 at day 7 and an insignificant median CI of only −0.14 at day 14. These data indicate that the gut colonization defects observed with F11 Δ*glpG* are not caused by disruption of *glpR* regulation but are instead attributable to the loss of *glpG* itself.

## DISCUSSION

Mucus is thought to be an important source of nutrients for ExPEC and other E. coli strains within the gastrointestinal tract. This assumption is primarily based on two observations: (i) commensal E. coli strains associate with the intestinal mucus layer in streptomycin-treated mouse models (15, 16), and (ii) the ability of a bacterial strain to utilize mucus for growth *in vitro* often correlates with increased bacterial fitness *in vivo* within the murine gut (16, 17). By comparing the numbers of individual mutants from our F11 transposon mutant libraries before and after incubations in M9 medium containing mucus (M9-mucus) broth versus M9 medium containing glucose (M9-glucose) broth, we aimed to identify loci that specifically promote ExPEC growth in mucus. Our Tn-seq screen highlighted 112 mucus-specific candidate fitness genes, along with 171 glucose-specific candidate fitness genes and 28 genes that seemed to promote ExPEC growth and survival in both types of broth (Fig. 1B; see also Data Set S1 in the supplemental material). Using specific targeted gene knockout strains, we validated the Tn-seq results for the mucus-specific genes *fadL*, *fbp*, and *glpG*, showing that each promotes ExPEC growth in mucus broth (Fig. 3B). In addition, we found that these three genes also facilitated ExPEC growth under conditions in which the LCFA oleate is the only carbon source (Fig. 3D and 5B). These results fit with the observation that a number of the candidate mucus-specific fitness genes that were identified by Tn-seq are known to be involved either directly or indirectly with fatty acid beta-oxidation (Fig. 1C).

The fact that ExPEC can utilize lipids while growing on mucus is not entirely unexpected. A previous study found that E. coli (including an ExPEC isolate) and Salmonella strains were capable of growing on lipids extracted from mucus, although the lipid fraction did not support bacterial growth to the same extent as whole mucus (40). In addition, transcriptional profiling indicated that growth of the E. coli K-12 strain MG1655 in mouse cecal mucus causes the induction of genes that are important for use of the lipid phosphatidylethanolamine (19). These studies support the notion that mucus-associated lipids, even though they are a relatively minor portion of the mucus layers within the gastrointestinal tract, are available for bacteria to metabolize.

Perhaps a more surprising result from our Tn-seq experiment was that few of the candidate mucus-specific fitness genes that were identified are linked with sugar utilization pathways. The mucin proteins that make up the bulk of mucus are heavily glycosylated with O-linked sugars (41) and could, in theory, be a rich source of energy for ExPEC. However, these sugars are likely inaccessible to ExPEC until they are released from mucins by activities associated with other members of the microbiota (14). In mucus broth, in the absence of other bacteria that can cleave O-linked sugars from mucins, lipids may be the primary nutrients that are readily available to ExPEC.

Of the three F11 mutants that had validated fitness defects in mucus broth, only the Δ*glpG* mutant was notably impaired in our mouse gut colonization assays (Fig. 6). This result is in line with previous findings showing that genes that are transcriptionally induced in E. coli during growth on mucus *in vitro* are not necessarily required for growth within the mouse intestinal tract (19). The partial disconnect between our *in vitro* and *in vivo* results may be attributable to variances in the availability of sugars in the different assays, as noted above, as well as the broader spectrum of environments that may be encountered by ExPEC within the gut. Specifically, the gut has multiple physically and nutritionally distinct niches that could conceivably support ExPEC colonization in a manner independent of mucus-derived nutrients. Evidence that multiple nutritional niches are available to E. coli within the gut comes from studies showing that different E. coli strains can simultaneously colonize the mouse intestinal tract if they can utilize distinct nutrients (42, 43). The extent to which two E. coli strains can coexist within the gut is dependent on the amount of overlap between their respective metabolic capabilities. If competition with other members of the microbiota is also considered, it is clear that there are selective pressures for ExPEC and other E. coli strains to have multiple ways to obtain nutrients within the gut, with the scavenging of mucus-derived products being only one strategy.

Another important consideration when assessing our results is that the mucus that was used in the broth culture assays differed from the mucus that is encountered by ExPEC within the mouse intestinal tract. While mucus is fairly consistent across mammalian species (41), there are notable variations in the composition of mucus from different sites within the body due to the expression of distinct mucin proteins and the elaboration of different glycosylation patterns. For example, our *in vitro* experiments utilized partially purified porcine gastric mucus, which mostly consists of the MUC5AC mucin protein (44). MUC5AC is primarily decorated with glycosylation moieties that are neutral, although some are monosulfated (45). In contrast, colonic mucus is largely comprised of the MUC2 mucin, a paralog of MUC5AC that is heavily modified with fucosylated glycans (44, 45). These differences could affect specific gene requirements for ExPEC in our *in vitro* versus *in vivo* assays. Nevertheless, results from our Tn-seq experiment have provided unique insight into how ExPEC can utilize mucus and survive within the gut and may also be relevant for understanding ExPEC colonization of mucosal tissues outside the intestinal tract. For example, during infection of the female genitourinary tract, ExPEC likely encounters mucus that is produced by the endocervix. This mucus is somewhat similar to the gastric mucus used in our Tn-seq experiment, being composed of MUC5AC and MUC5B mucins that are modified with a mix of neutral and acidic sugar side chains (46, 47). Consequently, it is feasible that some of the fitness genes that were highlighted by the Tn-seq experiment also contribute to ExPEC fitness within the genitourinary tract.

One of the primary goals of our Tn-seq screen was to identify genes that are important for ExPEC colonization of the gut by first defining loci that are critical for ExPEC growth in mucus. Despite various caveats to this approach, as noted above, we identified *glpG* to be a key regulator of both mucus metabolism and gut colonization by ExPEC. However, subsequent work showed that the *in vitro* defects associated with the Δ*glpG* mutant were due to polar effects on the downstream *glpR* gene, whereas the impaired ability of the mutant to colonize the gut was due to disruption of *glpG* itself. The former conclusion is supported by the observations that the Δ*glpR* mutant phenocopied F11 Δ*glpG* in the *in vitro* growth assays and that both mutants were rescued by plasmids expressing *glpEGR* but not *glpEG* (Fig. 3B). The lack of growth of any of the *glp* operon mutants on oleate is specifically attributable to diminished amounts of G3P (Fig. 5), an intermediate in the glycerol degradation pathway that is depleted when functional GlpR is missing (39). The depletion of G3P in the absence of functional GlpR may impair phospholipid biosynthesis, interfering with the creation of the lipid precursor phosphatidic acid from G3P and acyl-CoA molecules that are generated by beta-oxidation (48). This phenomenon may be especially detrimental when oleate is the sole carbon source that is available to ExPEC.

Within the mouse intestinal tract, the Δ*glpR* mutant did not phenocopy F11 Δ*glpG*, indicating that the disruption of *glpG* alone is sufficient to impair gut colonization by ExPEC (Fig. 6C and D). GlpG has been studied extensively as a model of the intramembrane rhomboid protease family, and much is known about its biochemical properties (49). However, to our knowledge, its natural substrate(s) is yet to be identified, and no *in vivo* phenotype has been associated with *glpG*. The biology of other members of the rhomboid protease family is better understood (50). For example, some rhomboid proteases in Drosophila function to release membrane-tethered epidermal growth factor receptor ligands, such as Spitz, Gurken, and Keren (51). Additional work is needed to identify the relevant substrate(s) of GlpG and to understand its role in gut colonization by ExPEC.

While the mouse gut colonization model used in this study allows for the functional analysis of ExPEC mutants like F11 Δ*glpG* in the presence of established, highly complex microbial communities, it does have some limitations. One of these is that the data variability increases over the course of each experiment, even when assessing mutants that exhibit no apparent colonization defect (see Fig. 6 and reference 22). This variability was not attributable to cage effects, as it was seen in multiple independent experimental replicates with mice that were separately housed. Other researchers have observed similar levels of variability in gut colonization experiments in which two E. coli strains of equal fitness were competed against one another (52, 53). In these studies, it was discovered that during colonization the bacteria acquired loss-of-function mutations at specific loci that led to increased fitness. These mutations give these experiments a dynamic nature, with different subpopulations derived from each of the competing strains expanding and contracting over time. It is likely that similar dynamics are at play in our mouse model, but additional experimentation is needed to confirm this possibility. Though the fluctuating fitness levels of competing strains due to spontaneous secondary mutations may obscure subtle mutant defects, our gut colonization model has proven useful for identifying mutants with more robust phenotypes that may arguably be of greater relevance for understanding ExPEC fitness within the intestinal tract (Fig. 6C) (22).

Cumulatively, our data indicate that ExPEC growth in mucus involves many gene products that are directly or indirectly associated with fatty acid degradation, but these genes are not necessarily critical for ExPEC colonization of the gut. Within the mouse intestinal tract, in the face of an intact microbiota, we found that ExPEC persistence is promoted by the rhomboid protease GlpG. This is an unusual instance in which a rhomboid protease significantly impacts bacterial fitness, opening up many questions concerning the functions of GlpG within the intestinal tract. A better understanding of GlpG and other bacterial factors that promote ExPEC growth and survival in the gut will provide a broader view of the ExPEC life cycle and may aid the development of new approaches to target ExPEC before it can disseminate into extraintestinal niches.

## MATERIALS AND METHODS

### Creation of bacterial strains and plasmids.

The cystitis isolate F11 was altered using strains carrying pKM208, which facilitates the creation of gene knockouts and knock-ins via bacteriophage lambda Red recombination (54). Knockout and knock-in constructs were created by PCR using either pKD3 or pKD4 as the template to amplify a chloramphenicol or kanamycin resistance cassette, respectively, along with ∼40 bp of flanking sequences with homology to the target genomic insertion sites (55). The strains used in this study, along with the primers used to create and verify the mutant bacteria, are listed in Table S1 in the supplemental material.

All expression plasmids utilized in this study were derived from pACYC177 (56) and are listed in Table S2 along with the primers used to create them. Each PCR product was ligated into pACYC177 using the BamHI and NheI sites, which also removed the kanamycin resistance cassette. The control plasmid pCWR40 was created by digestion of pACYC177 with BamHI and NheI, followed by a reaction with the Klenow fragment to create blunt ends prior to religation.

### Media.

While creating knockout, knock-in, and complemented strains, bacteria were grown in Luria-Bertani (LB) broth. All growth in petri dishes was done using LB agar supplemented with chloramphenicol (20 μg/ml), kanamycin (50 μg/ml), or ampicillin (100 μg/ml), as appropriate. When growing bacteria for mouse inoculation experiments, we utilized a modified M9 medium containing MgSO_4_·7H_2_O (1 mM), CaCl_2_·2H_2_O (0.1 mM), d-(+)-glucose (0.1%), nicotinic acid (0.00125%), thiamine HCl (0.00165%), Casamino Acids (0.2%), Na_2_HPO_4_ (6 g/liter), KH_2_PO_4_ (3 g/liter), NH_4_Cl (1 g/liter), and NaCl (0.5 g/liter), all of which were dissolved in water. For *in vitro* growth experiments in M9-glucose or M9-mucus broth, the above-described recipe was used, except that Casamino Acids were excluded. In addition, for the M9-mucus broth, 0.5% porcine gastric mucus (catalog number M1778; Sigma) was used in place of glucose. The mucus was dissolved in water by autoclaving for 20 min at 121°C.

### Mucus and glucose Tn-seq screens.

Three independently derived transposon mutant pools, which were described previously (21), were utilized in this experiment. Briefly, these mutant pools were created by conjugative transfer to F11 of the pSAM-Ec plasmid, which contains a himar1C9 transposase and a mariner transposon with a kanamycin resistance cassette. Transposon mutants were selected by plating on LB agar supplemented with kanamycin. Further analysis showed that few ampicillin-resistant bacteria were present, indicating that whole-plasmid insertion into the F11 chromosome occurred infrequently.

Each of the transposon mutant pools was expanded in LB broth and then subcultured 1:100 into M9-mucus or M9-glucose broth and grown at 37°C for 24 h with shaking at 180 rpm in a sealed jar containing a Mitsubishi AnaeroPack-MicroAero sachet. Each of these cultures was then subcultured 1:100 into fresh broth and grown for another 24 h under the same microaerobic conditions. After a total of 48 h of growth, cells were collected for DNA purification.

DNA was prepared for sequencing via homopolymer tail-mediated ligation PCR (HTML-PCR) as described previously (57), with some modifications. DNA from the input (LB broth) and output (M9-glucose and M9-mucus broth) samples was isolated using a Wizard genomic DNA purification kit (Promega). Thirty micrograms of genomic DNA from each sample was diluted in 50 μl of water and sonicated using a Diagenode sonicator with 30 cycles of 30 s on and 60 s off. This treatment led to sheared DNA being mostly less than 600 bp. The DNA was purified and concentrated using a GeneJet PCR purification kit (Fermentas), which removes DNA pieces below a size of 100 bp. Poly(dC) tails were added to the 3′ ends of the sheared DNA pieces by incubation of 1.5 μg of DNA with terminal deoxynucleotidyl transferase (TdT; NEB) at 37°C for 1 h, followed by heat inactivation at 75°C for 20 min. The products of the TdT reaction were purified using a DTR gel filtration cartridge (Performa) following the directions provided by the manufacturer. Two PCRs were performed in succession using the Easy A cloning enzyme. The first reaction mixture contained primers olj376 and tn_rev1-2 (Table S3), while the second one contained the primer tn_rev2-2 and an appropriate indexing primer. The products of the reactions were purified and sent for sequencing, using the pSAM-Ec Illumina seq primer, by the Tufts University Genomics Core Facility. Sequencing was done on a HiSeq 2500, high-output, v4, sequencer with single-end reads produced over 50 cycles.

### Bioinformatics.

Reads were initially processed using a custom Python script to remove adaptor sequences, trim low-quality bases from the 3′ end of the read (a minimum phred score of 20 was required), trim strings of C nucleotides that may have originated from the TdT reaction from the 3′ end, as well as filter out reads with low-quality 5′ ends (a minimum phred score of 20 was required). After trimming, any reads with a length of less than 28 nucleotides were removed. On average, these requirements led to ∼3% of reads being removed.

The trimmed reads were then aligned to the F11 genome using the Bowtie2 program, and the resulting SAM files were sorted using SAMtools. Custom Python scripts were used to analyze the sorted SAM files, including identification of transposon insertion locations and quantification of the number of reads for each insertion location. Lower-quality alignments were filtered out by requiring that each alignment have a Mapq score of at least 35 and no more than 2 mismatches and that it not align equally well to more than one location on the genome. Additionally, alignments were required to correspond to a TA dinucleotide, the preferred insertion location of the mariner transposon. These thresholds resulted in an additional ∼3% of the reads being filtered at this step. During this process, forward and reverse insertion events that occurred at the same location were pooled and considered one insertion site.

In order to calculate fitness scores for individual transposon insertion sites, a table that included the read counts for only those insertion sites that were detected in both samples was created for each input-output sample pair. Insertion sites were required to have at least 10 reads in the input sample to be included. Transposon mutants which were completely lost from the output sample were added to the table if the number of reads at that insertion site in the input sample was very high, within the top 1% of input sites. In this case, the output read count was set to 1, the limit of detection. This practice allowed for analysis of mutants with very low fitness under the growth conditions. The input-output pair tables were then normalized using geometric mean normalization by a strategy similar to the normalization strategy utilized by DESeq2 (58). The normalized reads were then used to calculate a fitness score for each insertion site in each sample, which was calculated as log_2_(output sample reads/input sample reads). The fitness scores for all of the insertion sites in a given gene across the three replicates were used to calculate a median fitness score, which was designated the fitness score for that gene (Data Set S1). A *P* value was calculated using a Wilcoxon signed-rank test, with the hypothetical median being set to 0.

A list of candidate genes that met certain criteria, which included a fitness score of −2.0 or lower (i.e., at least a 4-fold reduction in fitness from input to output) and a *P* value of 0.05 or lower (Data Set S1), was created. The candidate gene lists were analyzed using gene ontology via PANTHER (26).

### Competitive mouse gut colonization assays.

Mice were handled in accordance with protocols approved by the Institutional Animal Care and Use Committee at the University of Utah (protocol number 15-12015) following U.S. federal guidelines indicated by the Office of Laboratory Animal Welfare (OLAW) and described in the *Guide for the Care and Use of Laboratory Animals*, 8th ed. (59).

WT and mutant bacteria were grown statically in 20 ml modified M9 medium in a 250-ml flask for 24 h at 37°C. The WT and mutant strains were then mixed 1:1 (6 ml of each strain for a total of 12 ml) and pelleted by centrifugation at 8,000 × *g* for 10 min. After a wash with phosphate-buffered saline (PBS), the bacterial pellet was resuspended in 0.5 ml PBS. Adult female BALB/c mice were orally gavaged with 50 μl of the bacterial suspension containing a total of ∼1 × 10^9^ CFU. Mice, aged 7 to 8 weeks, were purchased from The Jackson Laboratory and housed at 3 to 5 mice per cage. The animals were allowed to eat (irradiated Teklad global soy protein-free extruded rodent diet) and drink water *ad libitum*. Feces were collected at the time points indicated above and in Fig. 6, homogenized in 1 ml of 0.7% NaCl, and briefly centrifuged at a low speed to pellet any insoluble debris. Supernatants were then serially diluted and plated onto LB agar plates containing either chloramphenicol (20 μg/ml) or kanamycin (50 μg/ml) to select for the relevant strains. Competitive indices (CIs) were computed as the ratio of mutant over WT bacteria divided by the ratio of mutant over WT bacteria in the inoculum.

### Bacterial growth on oleate plates.

To make M9-oleate plates, Triton X-100 (0.6%) and oleate (0.1%) were added to modified M9 medium (see above) that lacked Casamino Acids and glucose. The medium was rocked at room temperature for 20 min to facilitate mixture of the oleate with the Triton X-100. Molten agar was added to the mixture, and the plates were poured. Control plates were made by adding glucose (0.5%) or glycerol-3-phosphate (1 mM) instead of oleate. Once the plates were made, 1 μl of an overnight culture of each strain was streaked out and the plates were incubated at 37°C. Plates containing glucose were incubated for 24 h, whereas plates containing oleate or glycerol-3-phosphate as the only carbon source were incubated for 5 days.

### Statistics.

Statistical analysis for the Tn-seq experiment was performed using the NumPy package. All other statistical tests, as well as corrections for multiple comparisons using the Hochberg procedure (60, 61), were carried out using Prism (GraphPad) or Stata/IC-14 software.

### Accession number(s).

The Fastq files for the products of the sequencing reactions have been deposited in the Sequence Read Archive (SRA) under accession number SRP096298.

## Supplementary Material

Supplemental material
