# Supplementary material for: The Rhomboid Protease GlpG Promotes the Persistence of Extraintestinal Pathogenic Escherichia coli within the Gut
Source: Infect Immun. 2017 May 23;85(6):e00866-16. doi: 10.1128/IAI.00866-16 (PMC5442614; doi:10.1128/IAI.00866-16)
Supplement: Supplemental material [file supp_85_6_e00866-16__index.html]

The Rhomboid Protease GlpG Promotes the Persistence of Extraintestinal Pathogenic Escherichia coli within the Gut — Supplemental material 

# The Rhomboid Protease GlpG Promotes the Persistence of Extraintestinal Pathogenic Escherichia coli within the Gut

## Supplemental material

- Supplemental file 1 -

  Table S1. Bacterial strains used in this study. Table S2. Plasmids created for this study. Table S3. Primers used to prepare DNA for and perform Illumina sequencing.

  PDF, 106K
- Supplemental file 2 -

  Data Set S1. Tn-seq results/summary.

  XLSX, 608K
