# Supplementary material for: The Rhomboid Protease GlpG Promotes the Persistence of Extraintestinal Pathogenic Escherichia coli within the Gut
Source: Infect Immun. 2017 May 23;85(6):e00866-16. doi: 10.1128/IAI.00866-16 (PMC5442614; doi:10.1128/IAI.00866-16)
Supplement: Supplemental material [file IAI.00866-16_zii999092056s1.pdf]

Table S1. Bacterial strains used in this study. A list of the bacterial strains used in this work, along with the primers that were used to create them.

Strain	Description	Primers or Reference
F11::clm	F11 with a chromosomal insertion of a chloramphenicol resistance cassette at the intergenic region between genes *EcF11_2526* and *xseA*	1
F11::kan	F11 with a chromosomal insertion of a kanamycin resistance cassette at the intergenic region between genes *EcF11_2526* and *xseA*	F: TCTGGCGTAGCCTGGGAGTTATTGCCGGATGCGATGCTGGTGTGTAGGCTGGAGCTGCTTCG<br>R: TCACGTAAAAAAACGTCTAATCCGTAGACCGGATAAGAGGCATATGAATATCCTCCTTAG
F11Δ*appA*::clm	F11 derivative in which the *appA* gene has been replaced with a chloramphenicol resistance cassette	F: AATCAATAATGTCGGATATGAAAAGCGGAAACATATCGTGTGTAGGCTGGAGCTGCTTCG<br>R: TGGAGAGCGCACGCTGGTATGCGTGCTTCATTCACGATTT
F11Δ*fadL*::clm	F11 derivative in which the *fadL* gene has been replaced with a chloramphenicol resistance cassette	F: ATGAGCCAGAAAACCCTGTTTACAAAGTCTGCTCTCGCTGTGTAGGCTGGAGCTGCTTCG<br>R: CAGAACGCGTAGTTAAAGTTAGTACCGAACAGCCAGGCTTCATATGAATATCCTCCTTAG
F11Δ*fbp*::clm	F11 derivative in which the *fbp* gene has been replaced with a chloramphenicol resistance cassette	F: ATGAAAACGTTAGGTGAATTTATTGTCGAAAAGCAGCATGTGTAGGCTGGAGCTGCTTCG<br>R: TTACGCGTCCGGGAACTCACGGATAAAGCGTTCGACATCTCATATGAATATCCTCCTTAG
F11Δ*glpG*::clm	F11 derivative in which the *glpG* gene has been replaced with a chloramphenicol resistance cassette	F: TCCCCTTTTGTGTGGAATAAGCGACAGCAACGATGTTGTGTGTAGGCTGGAGCTGCTTCG<br>R: GTTGTGACGTTGTGTTTGTTTCATTTCTAAATCCCTGGAACATATGAATATCCTCCTTAG
F11Δ*glpR*::clm	F11 derivative in which the *glpR* gene has been replaced with a chloramphenicol resistance cassette	F: ATGAAACAAACACAACGTCACAACGGTATTATCGAACTCATATGAATATCCTCCTTAG<br>R: AGCACAGCTCCAGTTGAATATGATGGTCCTTCAGCACCTGTGTAGGCTGGAGCTGCTTCG
F11Δ*glpEGR*::clm	F11 derivative in which the *glpEGR* gene has been replaced with a chloramphenicol resistance cassette	F: ATGGATCAGTTCGAATGTATTAACGTTGCCGACGCGCACCCATATGAATATCCTCCTTAG<br>R: AGCACAGCTCCAGTTGAATATGATGGTCCTTCAGCACCTGTGTAGGCTGGAGCTGCTTCG

References:

(1) **Russell CW, Mulvey MA.** 2015. The Extraintestinal Pathogenic *Escherichia coli* Factor RqlI Constrains the Genotoxic Effects of the RecQ-Like Helicase RqlH. PLoS Pathog. 11(12):e1005317.

Table S2. Plasmids created for this study. A list of the plasmids that were created for this work, along with the primers used to create them.

Plasmid	Description	Primers
pCWR37	The *fadL* gene with native promoter from F11 inserted into BamHI and NheI sites of pACYC177	F: GGGCCGGATCCCGTTGATTTCCTCTGTATGTGC<br>R: GGGCCGCTAGCTCAGAACGCGTAGTTAAAGTTAGT
pCWR38	The *fbp* gene with native promoter from F11 inserted into BamHI and NheI sites of pACYC177	F: GGGCCGGATCCACGTCACTATCCTTTTTTAATCTGG<br>R: GGGCCGCTAGCTTACGCGTCCGGGAACTCA
pCWR39	The *glpEGR* operon with native promoter from F11 inserted into BamHI and NheI sites of pACYC177	F: GGGCCGGATCCGCTGCCCTCATTCACTTTCG<br>R: GGGCCGCTAGCTCAGCACAGCTCCAGTTGAA
pCWR40	pACYC177 was digested with BamHI and NheI, the sticky ends were blunted and ligated together.	Not Applicable
pCWR50	The *glpEG* genes with native promoter from F11 inserted into BamHI and NheI sites of pACYC177	F: GGGCCGGATCCGCTGCCCTCATTCACTTTCG<br>R: GGGCCGCTAGCTTATTTTCGTTTTCGCGCATTG

BamHI and NheI restriction sites are underlined.

Table S3. Primers used to prepare DNA for and perform Illumina sequencing. A list of the primers used to prepare genomic DNA for sequencing with the Illumina HiSeq, along with the custom sequencing primer.

Primer	Sequence	Reference
olj376	GTGACTGGAGTTCAGACGTGTGCTCTTCCGATCTGGGGGGGGGGGGGGGG	1
tn_rev1-2	GCATACGAAGACCGGGGACT	This study
tn_rev2-2	AATGATACGGCGACCACCGAGATCTACACTCTTTGCATACGAAGACCGGGGACT	This study
BC33	CAAGCAGAAGACGGCATACGAGATCGTGATGTGACTGGAGTTCAGACGTGTGCTCTTCCGATCT	1
BC34	CAAGCAGAAGACGGCATACGAGATACATCGGTGACTGGAGTTCAGACGTGTGCTCTTCCGATCT	1
BC35	CAAGCAGAAGACGGCATACGAGATGCCTAAGTGACTGGAGTTCAGACGTGTGCTCTTCCGATCT	1
BC37	CAAGCAGAAGACGGCATACGAGATCACTGTGTGACTGGAGTTCAGACGTGTGCTCTTCCGATCT	1
BC38	CAAGCAGAAGACGGCATACGAGATATTGGCGTGACTGGAGTTCAGACGTGTGCTCTTCCGATCT	1
BC39	CAAGCAGAAGACGGCATACGAGATGATCTGGTGACTGGAGTTCAGACGTGTGCTCTTCCGATCT	1
BC40	CAAGCAGAAGACGGCATACGAGATTCAAGTGTGACTGGAGTTCAGACGTGTGCTCTTCCGATCT	1
BC41	CAAGCAGAAGACGGCATACGAGATCTGATCGTGACTGGAGTTCAGACGTGTGCTCTTCCGATCT	1
BC50	CAAGCAGAAGACGGCATACGAGATCGTTTCACGTGACTGGAGTTCAGACGTGTGCTCTTCCGATCT	1
pSAM-Ec Illumina seq	GCATACGAAGACCGGGGACTTATCATCCAACCTGTTA	This study

(1) **Klein BA, Tenorio EL, Lazinski DW, Camilli A, Duncan MJ, Hu LT.** 2012. Identification of essential genes of the periodontal pathogen *Porphyromonas gingivalis*. BMC Genomics. 13:578
